# Structural insight into a glucomannan-type extracellular polysaccharide produced by a marine *Bacillus altitudinis* SORB11 from Southern Ocean

**DOI:** 10.1038/s41598-022-20822-3

**Published:** 2022-09-29

**Authors:** Urmi Halder, Koushik Mazumder, K. Jayaram Kumar, Rajib Bandopadhyay

**Affiliations:** 1 Microbiology Section, Department of Botany, The University of Burdwan, Burdwan, West Bengal 713104 India; 2 National Agri-Food Biotechnology Institute, Sector 81, SAS Nagar, Punjab, 140308 India; 3 Department of Pharmaceutical Sciences and Technology, Birla Institute of Technology, Mesra, Ranchi, Jharkhand 835215 India

**Keywords:** Polysaccharides, Genome informatics, Bacteriology

## Abstract

Extracellular polysaccharide (EPS) produced by a deep-sea, psychrotolerant *Bacillus altitudinis* SORB11 was evaluated by considering physiochemical nature and structural constituents. The productivity of crude EPS was measured ~ 13.17 g L^−1^. The surface topography of the crude EPS showed a porous, webbed structure along with a branched coil-like configuration. The crystalline crude EPS contained a high amount of sulfur. Further, the crude EPS was subjected for purification. The molecular weight of purified EPS was determined ~ 9.8 × 10^4^ Da. The purified EPS was appeared to show glucomannan-like configuration that is composed of → 4)-β-Manp-(1 → and → 4)-β-Glcp-(1 → residues. So, this polysaccharide was comparable to the structure of plant-derived glucomannan. Subsequently, EPS biosynthesis protein clusters like EpsC, EpsD, EpsE, and glycosyltransferase family proteins were predicted from the genome of strain SORB11, which may provide an insight into the production of glucomannan-type of polysaccharide. This low molecular weight linear form of glucomannan-type EPS might be involved to form a network-like unattached aggregation, and helps in cell-to-cell interaction in deep-sea microbial species.

## Introduction

Microbes inhabiting extreme environments often secret extracellular polysaccharides with distinctive features and these polysaccharides eventually protects microorganism from life-threatening conditions^1,2^. Extracellular polysaccharides with diverse functions participate in biofilm formation to construct a comfortable extracellular environment, adhesion, molecular recognition, intracellular signal transduction, and sometimes pathogenesis^3,4^. As a substantial component and a major part of reduced carbon reservoir in the ocean, extracellular polysaccharides influenced by altering the physical and biogeochemical microenvironment around the cells. For unattached aggregation in a marine environment, polysaccharides with proteins, lipids, and nucleic acids form an architectural matrix^4–10^. In an aqueous solution, exopolysaccharides showed their hydrophilic nature due to the presence of hydroxyl and carboxyl groups that confer a net negative charge, thus providing acidic properties^4,5^. Bacterial exopolysaccharides are found in different forms such as biofilms, aggregated in a matrix or in dissolved forms by forming weak interactions with other organic and inorganic materials that provide hydrogen bonding, electrostatic interactions, dispersion as well as cohesive forces. However, these weak interactions depend on the polymer size and frequency of the functional groups. Furthermore, cross-linking within adjacent polymer chains influenced by electrolyte concentration enables permanent attachment^11,12^. The hydrated extracellular polysaccharide matrix creates a buffer zone to provide stability in adjacent changing environments and helps in localization of secreted exoenzymes for the cycling of organic and inorganic materials^12,13^. Highly hydrated porous polysaccharide matrix acts as a sponge that traps nutrients in flowing liquids. High polyhydroxyl containing extracellular polysaccharides provide buffering in low-temperature, high-salinity^4,14,15^.

In the Southern Ocean region, highly concentrated and varied microbial communities amalgamate to form transparent exopolysaccharide particles (TEPs) that aggregate with other organic debris and produce larger particles known as “marine snow” that serves as a vertical exporter of fixed carbons from the euphotic zone to deep waters^4,16^. Bacterial extracellular polysaccharides in marine environment act as glue and form a fibrillar framework that provides opportunities for interactions with different free-living bacterial cells, as well as nutrient uptake. Extracellular polysaccharides produced by bacterial populations from particulate material of Southern Ocean are often decorated with sulfate, acetyl, and succinyl groups, all of these provide polyanionic quality due to the ionization of acidic groups at pH of seawater environment^4,17–19^.

The investigation of extracellular polysaccharides produced by bacterial communities from deep-sea ecosystems provided a glimpse of how biomolecules were stabilized in harsh environmental conditions in spite of their biotechnological interest^13,14,20–22^. Therefore, in this present study, we explored the morphological, and physical properties of crude extracellular polysaccharide (EPS) and the structural configuration of purified EPS produced by a deep-sea, psychrotolerant bacterium *Bacillus altitudinis* SORB11, which was previously reported from 3800 m deep Indian sector of the Southern Ocean^23^. Moreover, *In-silico* analysis of coding gene clusters responsible for EPS biosynthesis was also performed using the genome of strain SORB11.

## Results and discussions

### Production and characterization of EPS

**Figure 1:**
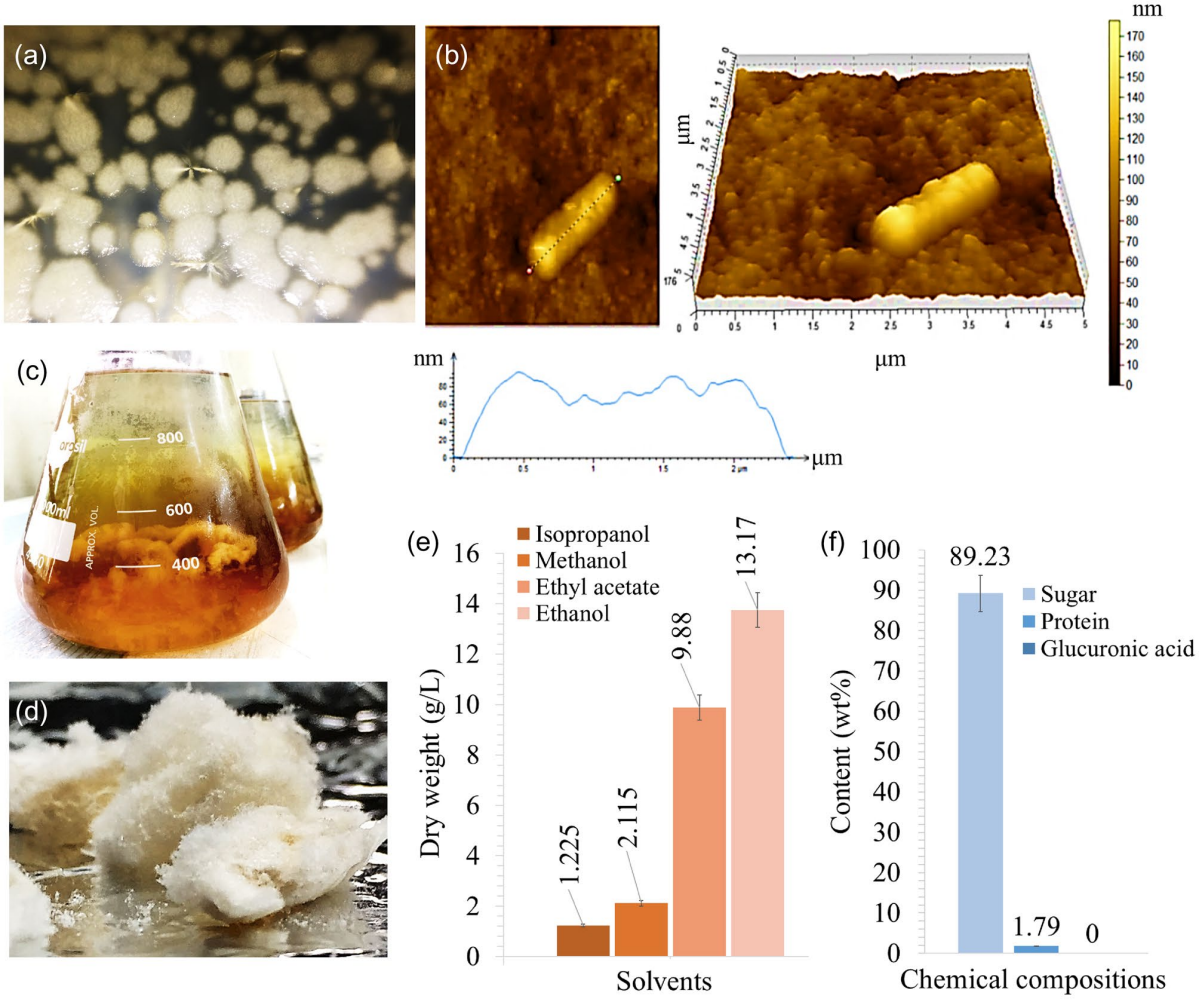
Mature colonies of *B*. *altitudinis* SORB11 (**a**); Atomic force micrographs showing cell morphology of strain SORB11 (**b**); EPS precipitation (**c**); lyophilized EPS powder (**d**); production optimization against different solvents (**e**) and chemical compositions (**f**) of the obtained crude EPS.

Free-living marine bacterial cells usually get aggregated with each other. Consequently, a fibrillar network is formed by secreting extracellular polysaccharides, and this ultrafine structure is assumed for cell-to-cell communication and to uptake nutrition from the environment^4,24^. A peculiar network formation (Fig. 1a) was observed between the colonies of *B*. *altitudinis* SORB11. Cells were rod-shaped bacilli, elongated with a diameter of ~ 1.0 µm and length of ~ 2.5 µm (Fig. 1b). Deposition of polysaccharide-like substances surrounding the cells (Fig. 1b) as well as polysaccharide precipitation was observed in the cell culture of strain SORB11 (Fig. 1c).

The extracted lyophilized EPS appeared as creamish white in color (Fig. 1d), it was soluble in polar solvents like water and dimethyl sulfoxide: dissolved ~ 1.0 g of crude EPS in 100 mL of water at 25 °C. Under optimum growth conditions (20 °C, pH level 8.00), ~ 13.17 g L^−1^ dry weight of crude EPS (Fig. 1e) was obtained using ethanol as a solvent. A total of 89.23% sugar component was present in the polysaccharide, with a 1.79% protein, and was devoid of glucuronic acid (Fig. 1f).

**Figure 2:**
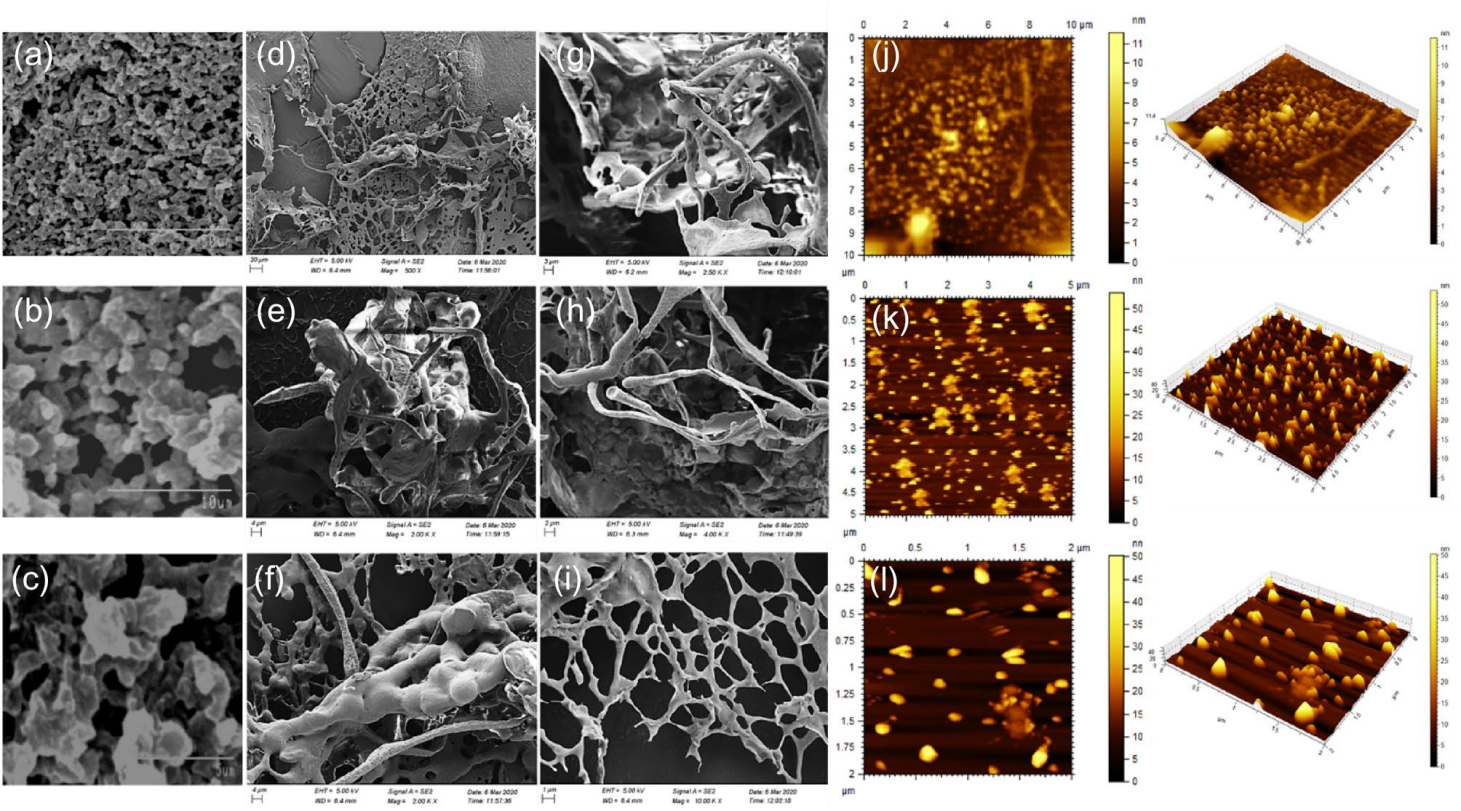
SEM images at magnifications 1.00 k × (**a**), 5.00 k × (**b**), and 10.00 k × (**c**) of lyophilized crude EPS; FE-SEM images at magnifications 500 × (**d**), 2.00 k × (**e**–**f**), 2.50 k × (**g**), 4.00 k × (**h**), and 10.00 k × (**i**) of 1.0 mg/mL crude EPS in solution; planar and 3-D AFM images representing 50.0 µg/mL (**j**), 10.0 µg/mL (**k**), and 5.0 µg/mL (**l**) concentrations of crude EPS in solution.

A heterogeneous type of surface morphology was exhibited by the crude EPS. The dried EPS powder showed porous surface topology in scanning electron micrographs (Fig. 2a–c). Whereas, a webbed configuration was mostly detected along with a long-branching patterns as well as, formation of coil-like structures of the EPS in solution at high magnification in Field emission scanning electron micrographs (Fig. 2d–i). In Atomic force micrographs (Fig. 2j–l), EPS showed ~ 4.3 µm long branching patterns at 50.0 µg/mL solution. But at 10.0 µg/mL and 5.0 µg/mL concentrations, the branched EPS was scattered and formed chain-like molecular structures (~ 200 nm long and ~ 10–25 nm diameter). Scattered molecules showed both impregnated as well as spherical surface topologies. Polysaccharides usually form disordered random coils with flexible chain-like configurations in an aqueous solution^25,26^. In marine environment, *B. altitudinis* SORB11 secrets a porous polysaccharide matrix that may helps to trap nutrients from flowing liquids. Moreover, adjacent polymer chains may cross-link between them to enable permanent attachment with the so-called “marine snow”^4^.

**Figure 3:**
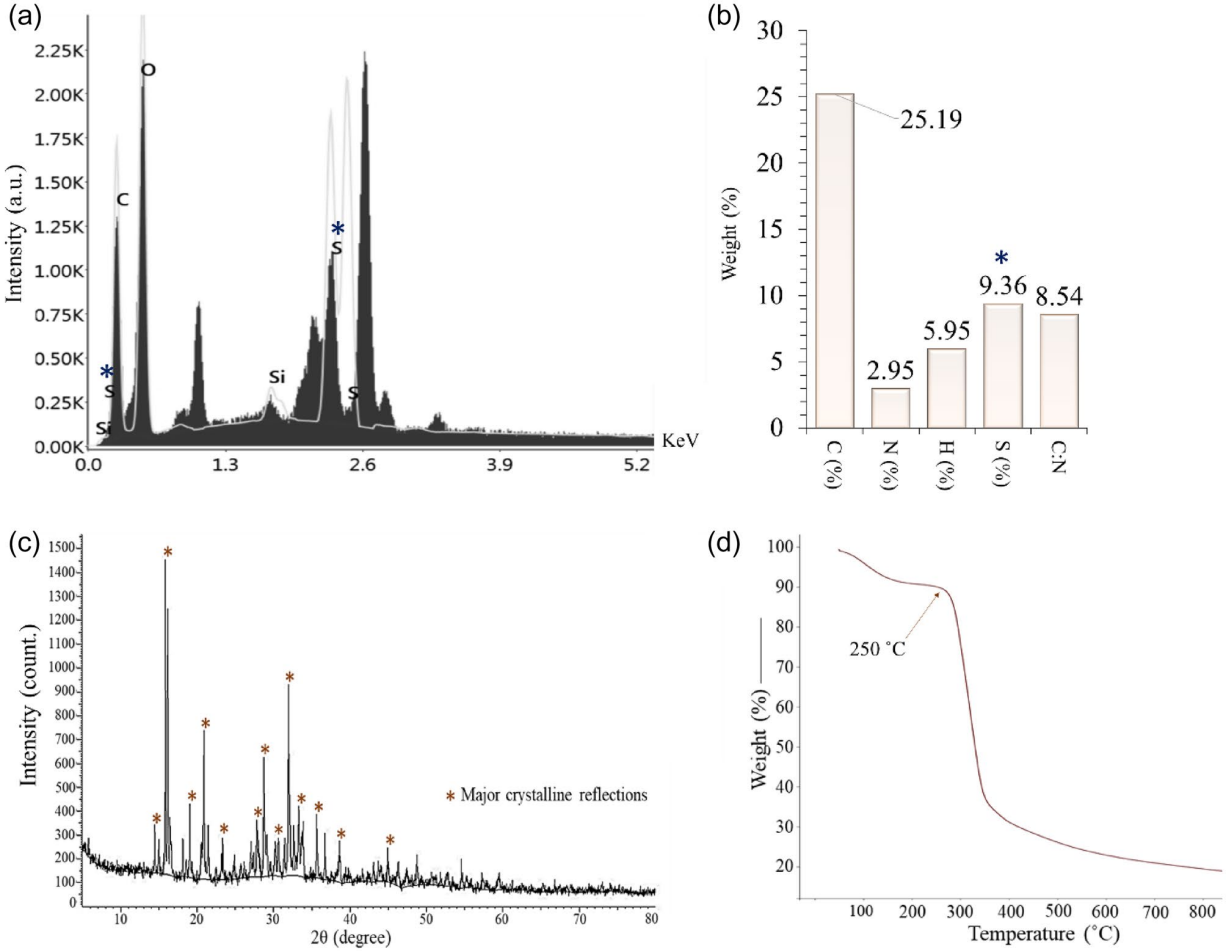
Study of EDX pattern (**a**), CNHS compositions (**b**), XRD pattern (**c**), and TG (**d**) of crude EPS.

The key non-metal elements of crude EPS were carbon, oxygen, and sulfur as revealed from EDX spectrum (Fig. 3a). The high content of sulfate was attributed to the peak at 168 eV which may responsible for the polyanionic nature of crude EPS^4,15^. That result was further confirmed by elemental analysis where with 25.19 w% carbon was the dominant element followed by 5.95 w% hydrogen. The presence of 2.95 w% nitrogen indicated the protein contamination which was confirmed earlier by the protein assay. The carbon/nitrogen ratio was 8.54. A fair amount of sulfur (9.36 w%) was also detected in the crude form of EPS (Fig. 3b).

X-ray diffraction pattern of crude EPS showed a major crystalline region from 15° to 40° (Fig. 3c). The diffraction pattern exhibited several intense narrow crystalline diffraction peaks at 16°, 21°, 29°, and 32° in the 2θ region that revealing the crystalline property of crude EPS^27^.

Thermogravimetric analysis was conducted dynamically among weight loss versus temperature (Fig. 3d). The EPS was stable over a wide range of temperatures. A major weight loss was recorded between 250 to 300 °C. So, the crude form of EPS was thermally stable up to ≥ 250 °C.

**Figure 4:**
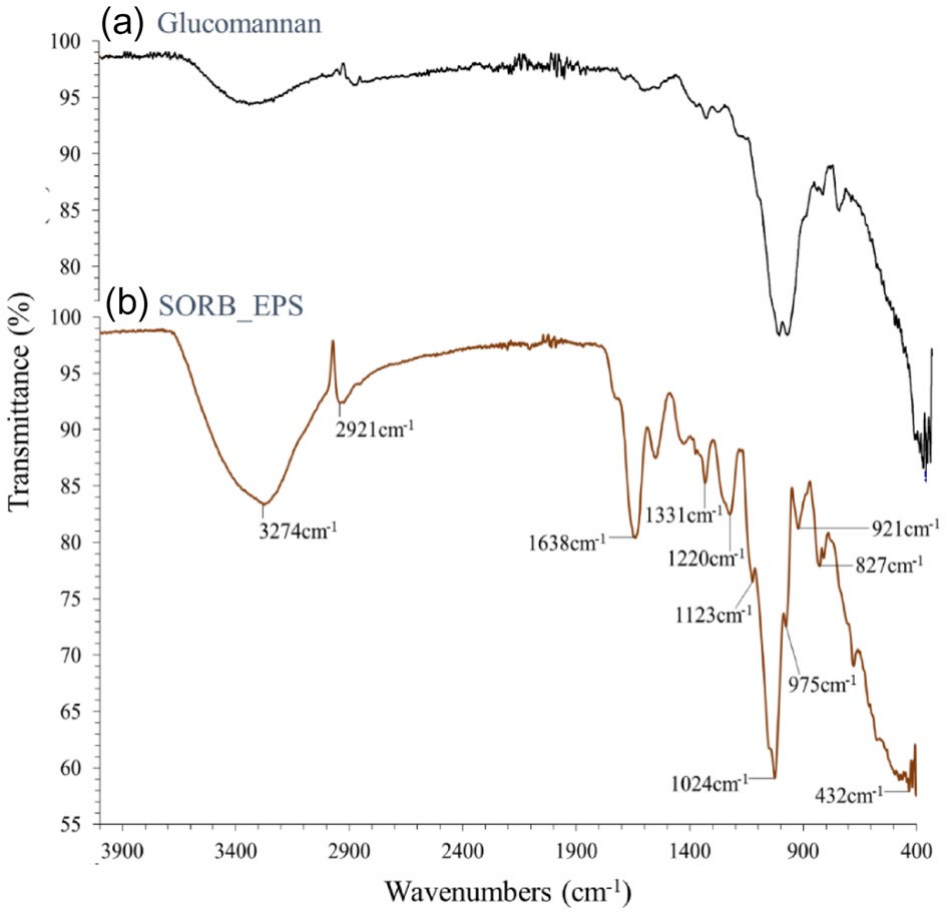
FT-IR spectra of standard glucomannan (**a**) and crude SORB-EPS (**b**).

A standard plant-derived glucomannan (Fig. 4a) and *B*. *altitudinis* SORB11 derived crude EPS (SORB-EPS) (Fig. 4b) were analyzed by IR spectra to detect functional groups and band assignments. For both polysaccharides, the absorption band that appeared in the range of 1000 − 1200 cm^−1^ indicated the presence of β-(1 → 4) linkage, but an adjacent peak in the range of 1031–1093 cm^−1^ that appeared only for glucomannan was due to the presence of (1 → 3) linked β-glucan^25,28,29^. Peaks at 3274 cm^−1^ and 2921 cm^−1^ were assigned to the O–H and C–H stretching and bending vibrations, respectively. The peak at 1638 cm^−1^ appeared due to the C=O stretching vibration of the protonated carboxylic acid or N-acetyl bonding^27,30^. Only in SORB-EPS, typical peaks that appeared at around 1220 cm^−1^ and 827 cm^−1^ were due to the stretching vibration of asymmetric S=O and symmetric C–O–S, respectively^17,31–33^. The presence of a significant amount of sulfur in SORB-EPS was previously confirmed by elemental and EDX analysis.

**Figure 5:**
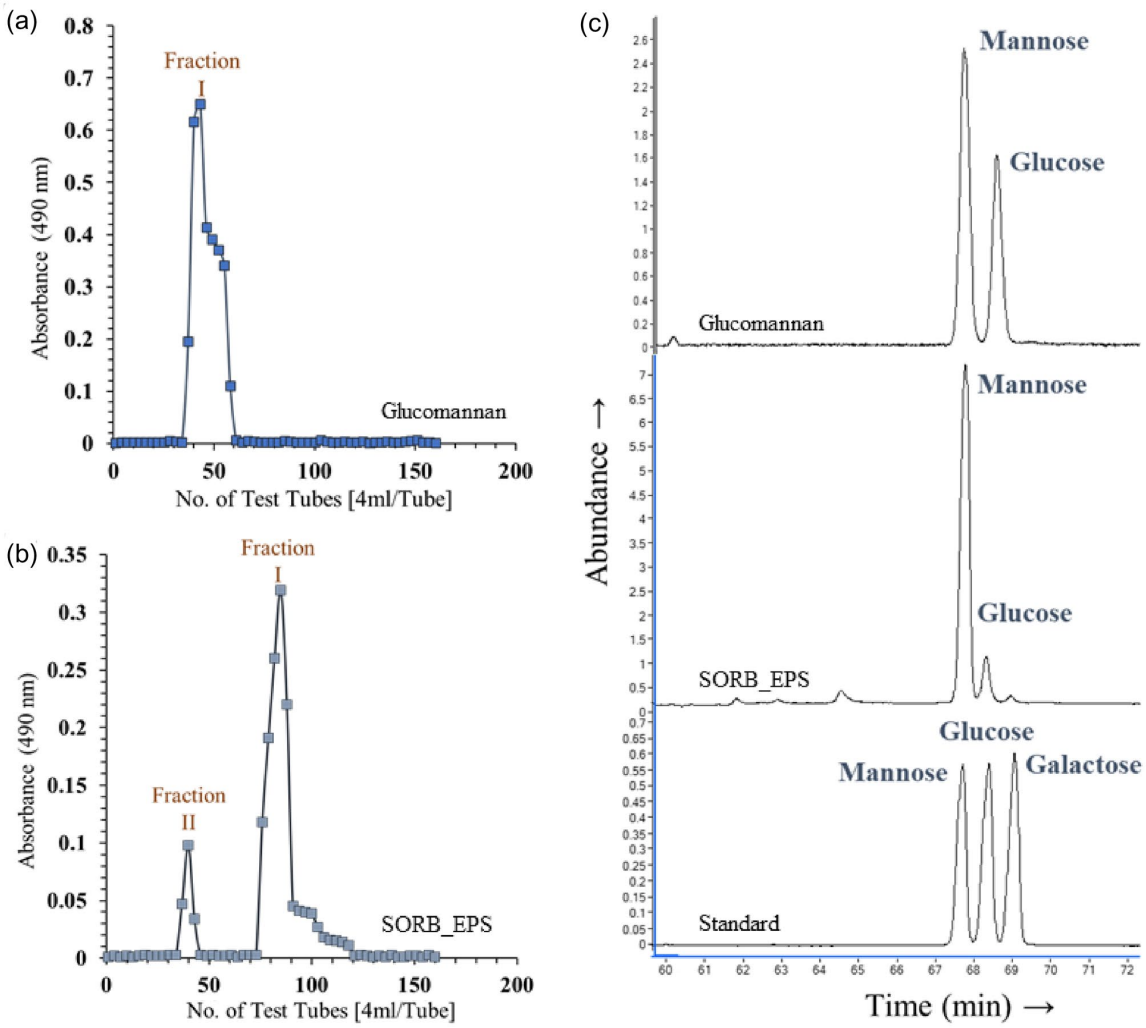
Gel permeation chromatography profile of standard glucomannan (**a**), SORB-EPS (**b**); and GC chromatograms of standard glucomannan, SORB-EPS, standard mannose, glucose, and galactose (**c**).

Crude SORB-EPS and standard glucomannan were purified with Gel-chromatographic technique. Fractions from major peaks were collected and lyophilized to get homogeneous polysaccharides in pure form (Fig. 5a, b). The molecular weight (Mw) of standard glucomannan was calculated ~ 1.50 × 10^6^ Da. Species of *Amorphophallus konjac* derived k-glucomannan generally represented with a Mw ~ 1.32 × 10^6^ Da^34^. Whereas, Mw of the major fraction *i.e.,* fraction-I of SORB-EPS was calculated to be ~ 9.8 × 10^4^ Da using dextrans as standard and was subjected for further characterization.

Monosaccharide analysis of standard glucomannan reflected the presence of mannose and glucose in a molar ratio of 1.64:1.00. Similarly, SORB-EPS showed a similar monosaccharide profile like glucomannan i.e., a heteropolysaccharide composed of mannose and glucose but with a different molar ratio of 8.86:1.00 (Fig. 5c). Therefore, the above results indicated the SORB-EPS was somewhat similar to that of plant-derived glucomannan^35^. However, it is an established fact that the ratio of mannose and glucose may vary depending on the origin of glucomannan^36^.

Methylation analysis of glucomannan represented 4-linked-D-mannopyranosyl and 4-linked-D-glucopyranosyl as the most abundant residues (Sup file 1). The presence of 3-linked-D-glucopyranosyl, as well as terminal residues, was also detected (Table 1). K-glucomannan derived from *Amorphophallus konjac* is a straight-chain polymer composed of β-(1 → 4)-linked D-mannose and D-glucose with a slide branching through β-(1 → 3)-glucosyl linkages^34^.

**Table 1:** Mass fragments of PMAA derivatives of glucomannan and SORB-EPS.

Type of sugar	PMAA	Deduced linkage	Molar ratio^a^	Retention time (min)	Massfragment
**Glucomannan**					
	2,3,4,6-Me_4_Man	T-Manp	1.00	49.61	71, 87, 102, 113, 118, 129, 145, 157, 162, 174, 190, 205
	2,3,4,6-Me_4_Glc	T-Glcp	0.16	50.36	71, 87, 102, 118, 129, 145, 162, 175, 205
Mannose	2,3,6-Me_3_Man	→ 4)-Manp-(1 →	1.84	53.61	71, 87, 102, 118, 129, 143, 162, 173, 190, 207, 233
Glucose	2,3,6-Me_3_Glc	→ 4)-Glcp-(1 →	1.36	53.99	71, 87, 99, 118, 129, 142, 159, 173, 190, 203, 233
	2,4-Me_2_Glc	→ 3)-Glcp-(1 →	1.13	54.38	71, 87, 101, 118, 129, 157, 174, 202, 217, 234
**SORB-EPS**					
	2,3,4,6-Me_4_Man	T-Manp	1.00	51.06	71, 87, 102, 113, 118, 129, 145, 157, 162, 174, 190, 205
Mannose	2,3,6-Me_3_Man	→ 4)-Manp-(1 →	8.16	53.25	71, 87, 102, 118, 129, 143, 162, 173, 190, 207, 233
Glucose	2,3,6-Me_3_Glc	→ 4)-Glcp-(1 →	1.94	53.88	71, 87, 99, 118, 129, 142, 159, 173, 190, 203, 233

PMAA, partially methylated alditol acetate.

^a^Molar ratios relative to the 3-linked-glucoseand 4-linked-mannose residues.

Likewise in the purified SORB-EPS, the most abundant residues were terminal and 4-linked-D-mannopyranosyl (Table 1). A 4-linked-D-glucopyranosyl residue was also identified but no such 3-linked branching pattern like plant-derived glucomannan was detected. In this way, the EPS produced by *B. altitudinis* SORB11 was linear and composed of β-(1 → 4)-linked D-mannose and β-(1 → 4)-linked D-glucose that described a simplified structure of glucomannan.

**Figure 6:**
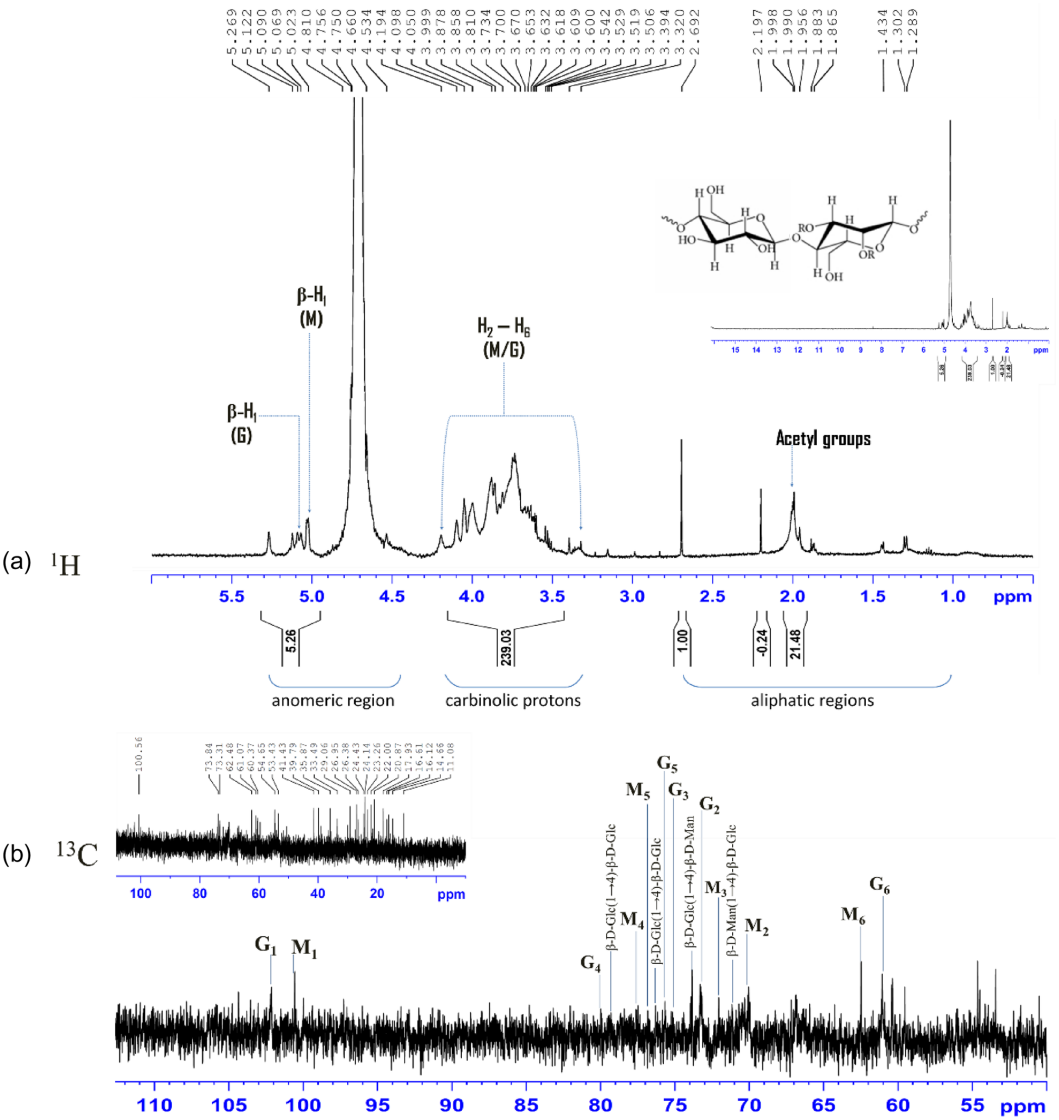
500 MHz proton (**a**), and carbon (**b**) NMR spectra of purified SORB-EPS.

The purified SORB-EPS was further subjected to proton and carbon NMR spectroscopy and the signals of mannose and glucose units according to their structures as well as the sequence of sugar units were compared with glucomannan stated in the literature^25,37–48^. Signals at 1.956 ppm, 1.990 ppm, and 2.197 ppm were assigned as methyl protons (CH_3_) of the acetyl group. Other signals like 5.090 ppm and 5.023 ppm were assigned as C-1-linked hydrogens of mannose and glucose, respectively. Overlapped signals from 3.302 to 4.098 ppm attributed to the hydrogens linked to C-2 to C-6 of both mannose and glucose. Signals at 4.194 ppm and 5.269 ppm indicated the position of acetyl groups at C-2, C-3, and C-6 like acetyl polysaccharides (Fig. 6a).

Carbon signals measurement was carried out with an overnight run, but S/N ratio was not that high may be due to the low concentration of the solution. The resonances at around 20.0 ppm to 25.0 ppm revealed the abundance of O-acetyl groups in SORB-EPS. The shifts at 103.0 ppm and 104.0 ppm were due to the C-1 anomeric resonance of 4-linked glucopyranosyl and at 101.0 ppm due to the C-1 anomeric resonance of 4-linked mannopyranosyl residues. The C-4 down-field shifts of 4-linked glucopyranosyl appeared at 77.0 ppm and 4-linked mannopyranosyl units at 79.0 ppm were due to the involvement of glycosidic linkages. The typical resonances of C-5, C-3, and C-2 of β-1, 4-linked glucopyranosyl residues were observed at 76.0 ppm, 75.0 ppm, and 74.0 ppm, respectively. The signals for C-5, C-3, and C-2 of 4-linked mannopyranosyl residues were observed at 76.0 ppm, 72.0 ppm, and 71.0 ppm, respectively. The resonances at C-6 of 4-linked glucopyranosyl and 4-linked mannopyranosyl residues were observed at 61.0 ppm and 62.0 ppm, respectively (Fig. 6b). So, the proton, as well as carbon signals of purified SORB-EPS, were similar to that of plant-derived glucomannan. However, for more structural clarification, homo and heteronuclear 2-D NMR spectroscopy are needed to perform further.

### Predicted EPS biosynthesis pathway

A total of 143 cell wall and capsule-related proteins were predicted in the genome of *B. altitudinis* SORB11, where 44 proteins belong to the capsular and extracellular polysaccharide part, 30 proteins belong to Gram-Positive cell wall components, and 69 uncategorized proteins. Under the capsular and extracellular polysaccharide part, genes were present that encode proteins for dTDP-rhamnose synthesis (4), CMP-N-acetylneuraminate biosynthesis (3), polysaccharide deacetylases (5), rhamnose containing glycans (10), exopolysaccharide biosynthesis (8), and sialic acid metabolism (14). For EPS biosynthesis, tyrosine kinase transmembrane modulator (EpsC), tyrosine kinase (EpsD) and phosphogalactosyl transferase (EpsE) proteins were predicted. An operon *yqxM*-*sipW*-*tasA* that positively regulates extracellular matrix biosynthesis was predicted. Moreover, a total of 26 glycosyltransferase family proteins were found in the genome of strain SORB11, viz*.,* multispecies glycosyltransferase family 1 (1), bifunctional and multispecies glycosyltransferase family 2 (7), glycosyltransferase family 4 (2), glycosyltransferase family 39 (2), multispecies WecB/TagA/CpsF family glycosyltransferase (1), and multispecies glycosyltransferase (13) proteins.

Usually, for the regulation of EPS biosynthesis, interactions occur with the gene products homologous to *epsCDE*. In the initial step of EPS biosynthesis, EpsE is required. But for the activation of EpsE, functional EpsC and EpsD proteins are necessary where EpsC is required for the phosphorylation of EpsD. So, the activity of EpsE regulation by EpsC and EpsD possibly interacts in a complex to enable the biosynthesis of repeat units^49,50^.

**Figure 7:**
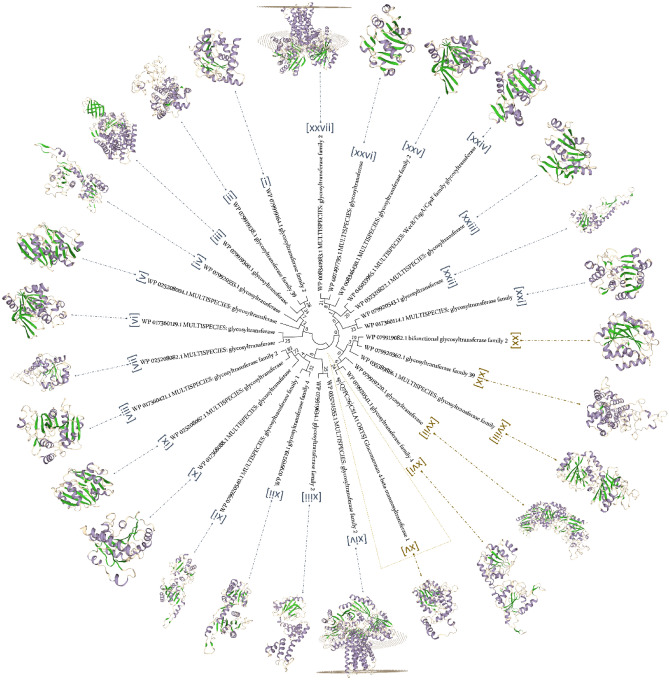
Phylogenetic tree of glycosyltransferase family proteins in the genome of strain SORB11 and OsCSLA1 protein from rice plant with their respective model (homology model) using minimum evolution with the close neighbor interchange inference method in MEGA 7.

Heteropolysaccharide biosynthesis starts with the formation of intracellular EPS precursors that serve as the donor monomers for most of the repeating unit biosynthesis. The monomeric units of the EPS backbone are transferred by glycosyltransferases and facilitate the formation of glycosidic linkages between them^51–53^. Two types of glycosyltransferase enzyme activity are reported for the biosynthesis of glucomannan, mannosyltransferase which utilizes GDP-D-mannose and glucosyltransferase which utilizes GDP-D-Glucose as a substrate^54,55^. Here, a well-studied OsCSLA1 protein sequence that basically incorporates glucose into glucomannan from the donor substrate GDP-glucose^54,56^ was compared with the glycosyltransferase family protein clusters of strain SORB11 (Fig. 7). OsCSLA1 protein clustered with glycosyltransferase family 39 (functions as 4-amino-4-deoxy-L-arabinose transferase or related glycosyltransferases of PMT family); glycosyltransferase family 4 (functions as GDP-mannose-dependent α-1-2-phosphatidylinositol mannosyltransferase); bifunctional glycosyltransferase family 2 (functions as polypeptide N-acetyl galactosaminyltransferase); glycosyltransferase (functions as UDP-n-acetylglucosamine-n-acetylmuramyl-(pentapeptide) pyrophosphoryl-undecaprenoln-acetylglucosamine transferase); and multispecies of glycosyltransferase family protein (functions as ATP synthase subunits region ORF 6, the UDP complex structure of the sixth gene product of the F1-ATPase operon). So, this set of proteins along with other glycosyltransferases (Sup file 2.) may responsible for the biosynthesis of glucomannan type of extracellular polysaccharide in strain SORB11.

**Figure 8:**
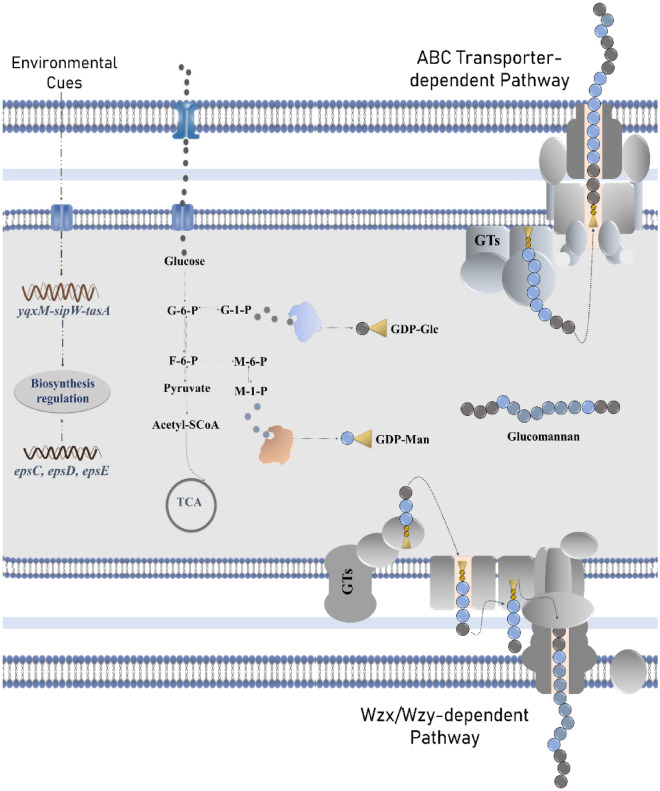
Schematic representation of probable EPS biosynthesis pathway based on the predicted coding genes from the genome of strain SORB11.

As per the previous report, there are four types of generalized transport mechanisms for the biosynthesis of polymers in bacterial system. However, ABC transporter dependent as well as, Wzx/Wzy dependent pathway which assembled by various glycosyltransferases are mainly responsible for the biosynthesis of heteropolysaccharides^57,58^. The different steps of a probable EPS biosynthesis pathway were stated according to the coding genes present in the genome of strain SORB11 (Fig. 8).

## Discussion

Marine bacteria generally produce linear heteropolysaccharides that are arranged in clusters of 10 or fewer repeating units of pentoses (arabinose, ribose, xylose), hexoses (glucose, mannose, galactose, allose, rhamnose, fucose), amino sugars (glucosamine, galactosamine), and uronic acids (glucuronic acid, galacturonic acid) with an average Mw ranging from 1 × 10^5^ to 3 × 10^5^ Da^4–6^. Species of *Vibrio*, a heterotrophic facultative anaerobe, isolated from extreme deep-sea of East Pacific Rise produced novel extracellular polymers that composed of heteropolysaccharides with uronic acids and amino sugars, and trace neutral sugars. The species of *Pseudoalteromonas* reported from particulates of Antarctica and Southern Ocean produced sulfated heteropolysaccharides with uronic acids, acetyl, and succinyl groups; whereas, *Pseudoalteromonas* species isolated from the deep-sea of Guaymas Basin produced sulfated (6–13%) heteropolysaccharides with high uronic acids, pyruvate, and acetate groups^13^. The species of *Alteromonas* isolated from 2600 m depth of East Pacific Rise produced high molecular weight deepsane type of extracellular polysaccharide^11,59^.

Here, *B. altitudinis* SORB11 produced low molecular weight thermally stable sulfur containing heteropolysaccharides. The capsular polysaccharide (CPS) of strain SORB11 was also composed of d-mannose, d-glucose, d-galactose^60^. The sulfur content within both extracellular and capsular polysaccharides of strain SORB11 is the indication of its marine origin^4^. The EPS was a linear and simplified structure of plant-derived glucomannan. The genome of strain SORB11 contained protein coding genes corresponding to EPS production. So, these gene clusters are likely to interact in a complex, enabling the biosynthesis of polysaccharide repeat unit.

Other way, biofilms are multicellular aggregates composed of various combinations of DNA, protein, and polysaccharides and are stabilized by an extracellular matrix. In the marine ecosystems, biofilm formation takes place when planktonic bacteria start to secret exopolysaccharide matrix particularly to attach themselves with a surface^61,62^. The transition of biofilm formation is governed by a series of complex regulatory proteins. In the genome of strain SORB11, some of the genes were significantly predicted for exopolysaccharide matrix formation: for example, *remA* gene encoding multispecies of extracellular matrix/biofilm regulator protein RemA, *ahpA* gene encoding multispecies of biofilm-specific peroxidase enzyme AhpA, and multispecies of SipW-dependent-type signal peptide-containing protein. In *Bacillus subtilis*, two small proteins RemA and RemB were appeared to act in parallel like other biofilm regulators, SinR, AbrB, DegU were found to involve for the activation of matrix biosynthesis operons. Exopolysaccharide and TasA protein are the two primary structural components of an extracellular matrix assembly. The signal peptidase SipW encoded by *yqxM* operon is required for TasA secretion and localization to the matrix. Moreover, AhpA is a peroxidase enzyme that is expressed during biofilm formation, protecting cells within biofilms by scavenging free radicals^62,63^. The capsular polysaccharide of strain SORB11 showed a significant level of free radical scavenging activities^60^. So, the extracellular part i.e.*,* EPS would expect to exhibit a satisfactory level of scavenging potentialities.

## Conclusion

In this study, the nature and chemical structure of EPS produced by a deep-sea inhabiting *B*. *altitudinis* SORB11 were explicitly stated. It was a novel low molecular weight glucomannan type of EPS having mannose and glucose. The genome also represented the presence of glucosyltransferase and the other needful family proteins of glycosyltransferase for the biosynthesis of glucomannan. This low molecular weight microbial glucomannan act as a connector by forming networks surrounding the cells and moreover, it was hypothesized that this structure might help in cell-to-cell communication and supply nutrition under stressful conditions. However, investigations on the microbial glucomannan containing sulfur would expect to unfold the complete nature and functions of the unusual network formed by marine, free-living bacteria for survival under floating conditions.

## Material and methods

### Isolation of strain SORB11

The water sample was collected aseptically using a rosette conductivity temperature depth (CTD) instrument (Sea-Bird Electronics, Inc. Florida, USA) from the Indian Sector of Southern Ocean (50°S, 47°E; Station No. 22) in austral summer (January–March, 2011) during the 5th Southern Ocean Expedition conducted by National Centre for Antarctic and Ocean Research (NCAOR) and Ministry of Earth Sciences (MoES), Government of India. Water sample was collected from 3800 m depth. During the sampling period, sea surface temperature was 5.5 °C, depth chlorophyll maxima was 55 m and atmospheric pressure was 1019 hPa. Strain SORB11 was isolated, cultivated in Zobell Marine Broth 2216 (ZMB, HiMedia) medium at 20 °C and identified as *Bacillus altitudinis*^23^*.*

### Production and optimization of crude EPS

Freshly prepared ~ 10.0 µL culture from exponential growth phase (Log CFU/mL = 6.60) of strain SORB11 was inoculated and cultivated on 1.0 L ZMB medium at 20 °C for 7 days at 150 rpm to increase the cell mass. The cell culture was then centrifuged at 5000×*g* for 20 min at 4 °C. For the removal of protein part, 5% (w/v) trichloroacetic acid (Sigma-Aldrich) was added with the cell-free supernatant and incubated overnight at 4 °C. The suspension was then centrifuged at 12,000×*g* for 20 min at 4 °C. An equal volume of 90% chilled ethanol (Sigma-Aldrich) was added with the supernatant and incubated overnight at 4 °C. The precipitated EPS part was collected by centrifugation at 12,000×*g* for 20 min at 4 °C. The pellet was washed with acetone two times and freeze-dried. The dry weight was calculated of that lyophilized EPS powder and stored at 4 °C for further analysis^30^. In order to get maximum EPS production under optimum growth conditions, in the precipitation step, different solvents like methanol (Sigma-Aldrich), isopropanol (Sigma-Aldrich), and ethyl acetate (Sigma-Aldrich) were used instead of ethanol.

### Characterization of EPS

#### Chemical analysis

Carbohydrate estimation was performed to determine the total sugar content using glucose (Sigma-Aldrich) as standard^64^. Lowry method using Bovine Serum Albumin (BSA, Sigma-Aldrich) as a standard^65^ was performed to detect the total protein content. The Carbazole method was performed to determine glucuronic acid^66^.

#### Solubility test

For the dissolution of EPS, 1.0 mg powder was mixed with 3.0 mL of various non-polar and polar solvents like chloroform (Sigma-Aldrich), trifluoroethanol (Sigma-Aldrich), dimethyl sulfoxide (Sigma-Aldrich), acetic acid (Sigma-Aldrich), and water. The mixtures were properly vortexed and solubilities were checked^15^.

#### Surface topography study

Scanning electron microscopy (SEM, S530 Hitachi) was performed with lyophilized EPS. Then, 1.0 mg/mL EPS solution using Milli-Q was prepared, drop casted on the stab and air-dried. Field emission scanning electron microscopy (FE-SEM, Zeiss Gemini 300, Germany) of that dried, gold-coated (IB2 ion coater) EPS sample was performed with an accelerating voltage of 5.00 kV. Further, 50.0, 10.0, and 5.0 µg/mL of crude EPS solutions were prepared using Milli-Q and 1.0 µL of each EPS solutions were drop casted onto a freshly cleaved mica surface. The sample was then air dried at ambient humidity and temperature for 2 hours and the micrographs were taken with an Atomic force microscope (AFM, 5500 Agilent Technologies, USA) at tapping mode. The cell morphology of strain SORB11 was also observed under the AFM^25^.

#### Elemental analysis

The quantification of C, H, N, S, and C/N ratio was determined with a CHNS analyzer (Vario EL III, M/s Elementar, Germany). A ~ 5.0 mg of crude EPS powder was mixed with vanadium pentoxide oxidizer in a tin capsule (1000 °C reactor) to perform the analysis. Further, elements like C, H, N, and S content of the crude EPS were measured with an Energy-dispersive X-ray spectroscope (EDS, INCA 250, Oxford Instruments, UK) of EPS was performed using a microprobe.

#### Physical property study

The physical structural property of crude EPS powder was measured with an X-ray diffractometer (XRD, PANalytical X'pert PRO 2200, Malvern Instruments, UK) equipped with Cu radiation at 40 mA and 40 kV. The X-ray diffraction pattern was recorded from 5.00° to 80.00° at a 2θ angle.

#### Thermal stability analysis

Thermogravimetric analysis with ~ 5.0 mg of crude EPS powder was carried out using TG/ DTA (Pyris Diamond, Parkin Elmer, USA). The sample weights and the signals were recorded from 10 to 800 °C temperature range under a nitrogen atmosphere using alumina as a control with a heating rate of 10 °C/min.

#### Infrared spectroscopy

Fourier transform infrared spectroscopy (FT-IR, IR-Prestige 21, Shimadzu, Japan) of strain SORB11-derived EPS, i.e., represented as SORB-EPS and a plant-derived glucomannan (Now Foods) as a standard were carried out for the detection of functional groups. The IR spectra were recorded at room temperature with a resolution of 1 cm^−1^ in 400–4000 cm^−1^ using KBr pellet method.

#### Purification of crude EPS and molecular weight determination

Size-exclusion based gel permeation chromatography (GPC) was performed using Sepharose-6B (GE Healthcare) column (90 cm × 2.1 cm) to purify both SORB-EPS and glucomannan. A ~ 25 mg polysaccharide sample was dissolved in 5.0 mL deionized water and passed through a 0.22 µ filter. The total volume of the filtrate was loaded on the top of the column. Deionized water was used as eluent with a flow rate of 0.1 mL/s. In each tube, 4.0 mL of eluent was collected and measured spectrophotometrically (UV-2600, Shimadzu, Japan) by using phenol-H_2_SO_4_ method at 490 nm. Finally, the fractions containing sugar were collected and lyophilized to get the purified polysaccharides. Molecular weight of SORB-EPS and glucomannan were also determined by these Gel-chromatographic techniques using T-40, T-60, T-70, T-200, and T-250 species of dextran (Sigma-Aldrich) as standard^67,68^.

#### Monosaccharide composition analysis of purified EPS

For the analysis of monosaccharide component, alditol acetate derivatization was performed using 1.0 mg of lyophilized polysaccharide hydrolyzed with 2 M trifluoracetic acid (Sigma-Aldrich) at 120 °C for 2 hours followed by a further reduction using Sodium Borohydride (NaBD_4_, Sigma-Aldrich)_,_ and acetylation was performed with acetic anhydride (SD Fine chemicals) and pyridine (Sigma-Aldrich) (1:1) at 80 °C for 20 minutes^69,70^. 1.0 µL derivatized monosaccharides were injected into the Gas Chromatography coupled with a mass spectrometer analyzer (Agilent Technologies 7890, USA) using helium as carrier gas (temperature gradient was 80 °C for 2 min, 80–170 °C at 30 °C/min, 170–240 °C at 4 °C/min, 240 °C for 30 min and ionized by electrons impact at 70 eV).

#### Glycosyl linkage analysis of purified EPS

The composition of glycosyl linkage of SORB-EPS and standard glucomannan were analyzed by the generation of partially methylated alditol acetate derivatives^71–73^. ~ 2–3 mg samples were dissolved in 2.0 mL Dimethyl Sulfoxide Anhydrous (DMSO, Sigma-Aldrich) and ~ 15.0 mg freshly prepared Sodium Hydroxide (NaOH, Sigma-Aldrich) was added and the reaction mixtures were allowed to stand for 15 minutes at room temperature. 1.0 mL iodomethane (Sigma-Aldrich) was added to each sample and stirred for 30 minutes at room temperature. After removing excess iodomethane, washed and dried methylated samples were further derivatized into partially methylated alditol acetate and analyzed by Gas Chromatography coupled with a mass spectrometer analyzer (Agilent Technologies 7890, USA) as described in “monosaccharide composition analysis” section. The GC EI-MS spectra of the partially methylated alditol acetate (PMAA) derived from a glycosidically linked sugar residue were identified using The CCRC Spectral Database for PMAA’s.

#### Nuclear magnetic resonance spectroscopy of purified SORB-EPS

For ^1^H and ^13^C NMR spectroscopy, ~ 15.0 mg purified SORB-EPS was dissolved in 0.5 mL D_2_O (99.9%, Sigma-Aldrich). The spectra were recorded on a 500 MHz NMR spectrometer (Bruker, USA) with a 5 mm inverse probe at 25 °C sample temperature. Acetone (H 2.225 ppm, C 30.4 ppm) was used as an internal standard^71,72^.

### Prediction of probable EPS biosynthesis pathway

The coding gene clusters responsible for EPS biosynthesis were annotated from the draft genome of strain SORB11^23^ from NCBI-PGAP^74^ and RAST^75^. OsCSLA1 protein^54^ (sp|Q7PC76|CSLA1_ORYSJ) was retrieved from NCBI-GenBank database. Glycosyltransferase family proteins responsible for EPS biosynthesis were analyzed by building homology model using SWISS-MODEL^76^ and functions from CAZy database^77^. Phylogenetic relationships and similarity index were studied by constructing a tree using minimum evolution with the close neighbor interchange inference method and 1000 bootstrap replicons in MEGA 7^78^.

## Supplementary Information

Supplementary Information 1.

Supplementary Information 2.

## Data Availability

Whole-genome shotgun project of SORB11 was deposited at DDBJ/ENA/GenBank under the accession number MEHW00000000; BioProject number PRJNA341563; BioSample number SAMN05726035 (https://www.ncbi.nlm.nih.gov/assembly/GCA_002042895.1). OsCSLA1 protein sequence is available in the NCBI-GenBank database (https://www.ncbi.nlm.nih.gov/gene/4328585).
