# Supplementary material for: Structural insight into a glucomannan-type extracellular polysaccharide produced by a marine Bacillus altitudinis SORB11 from Southern Ocean
Source: Sci Rep. 2022 Sep 29;12:16322. doi: 10.1038/s41598-022-20822-3 (PMC9523031; doi:10.1038/s41598-022-20822-3)
Supplement: Supplementary file 1 — Supplementary Information 1. [file 41598_2022_20822_MOESM1_ESM.docx]

**Supplementary File 1.** Linkage analysis of SORB_EPS and standard glucomannan


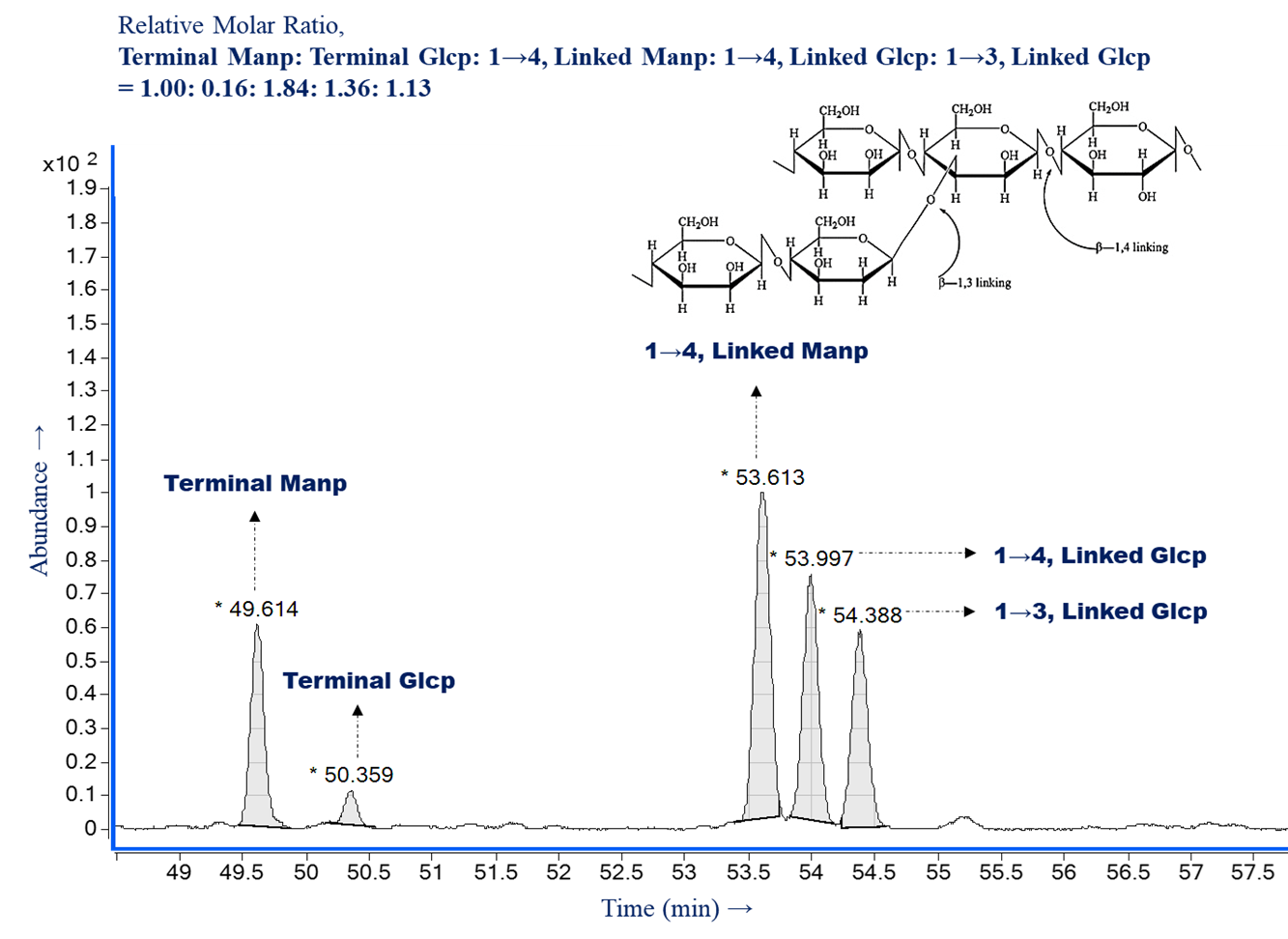


**Sup Fig 1.** Gas chromatography-mass spectrometry chromatograms of methylated Glucomannan


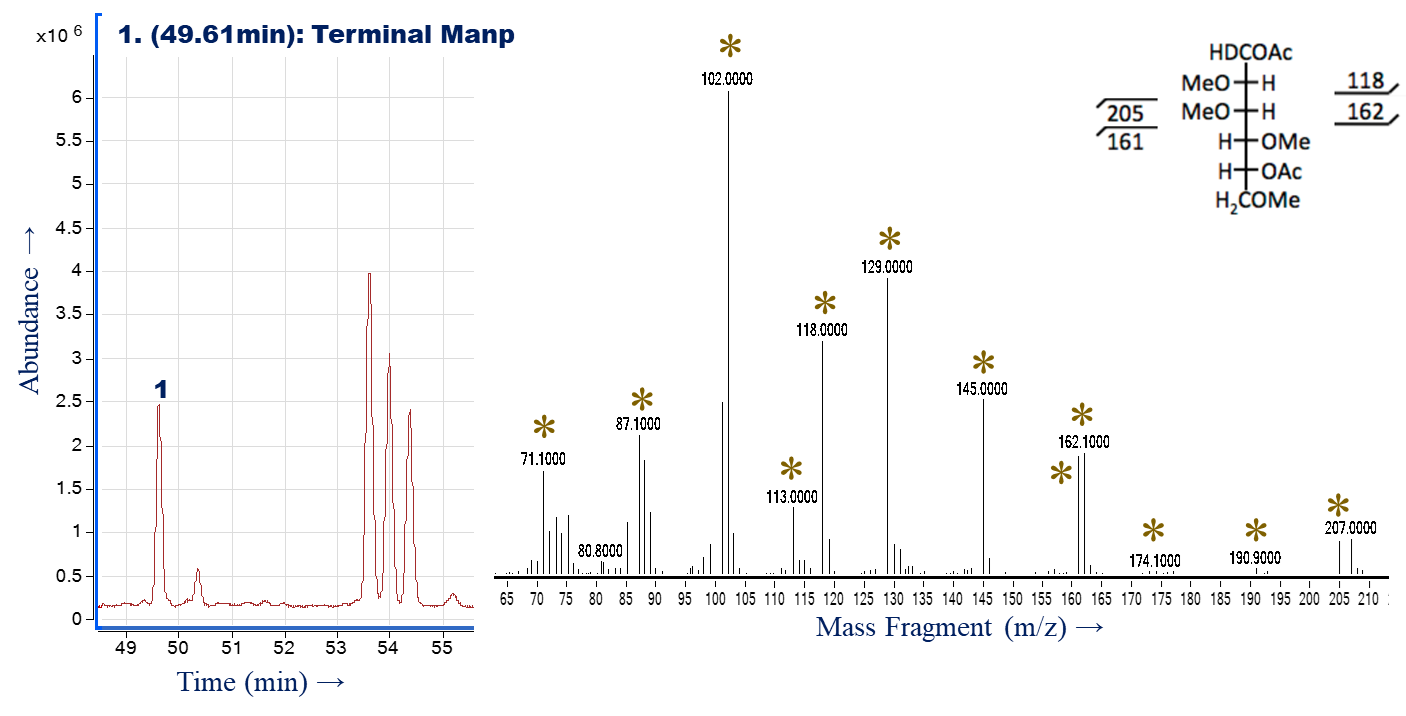


**Sup Fig 2.** The PMAA derivative of terminal-D-mannopyranosyl residue [1,5-Di-O-acetyl-1-deuterio-2,3,4,6-tetra-O-methyl-D-mannitol]


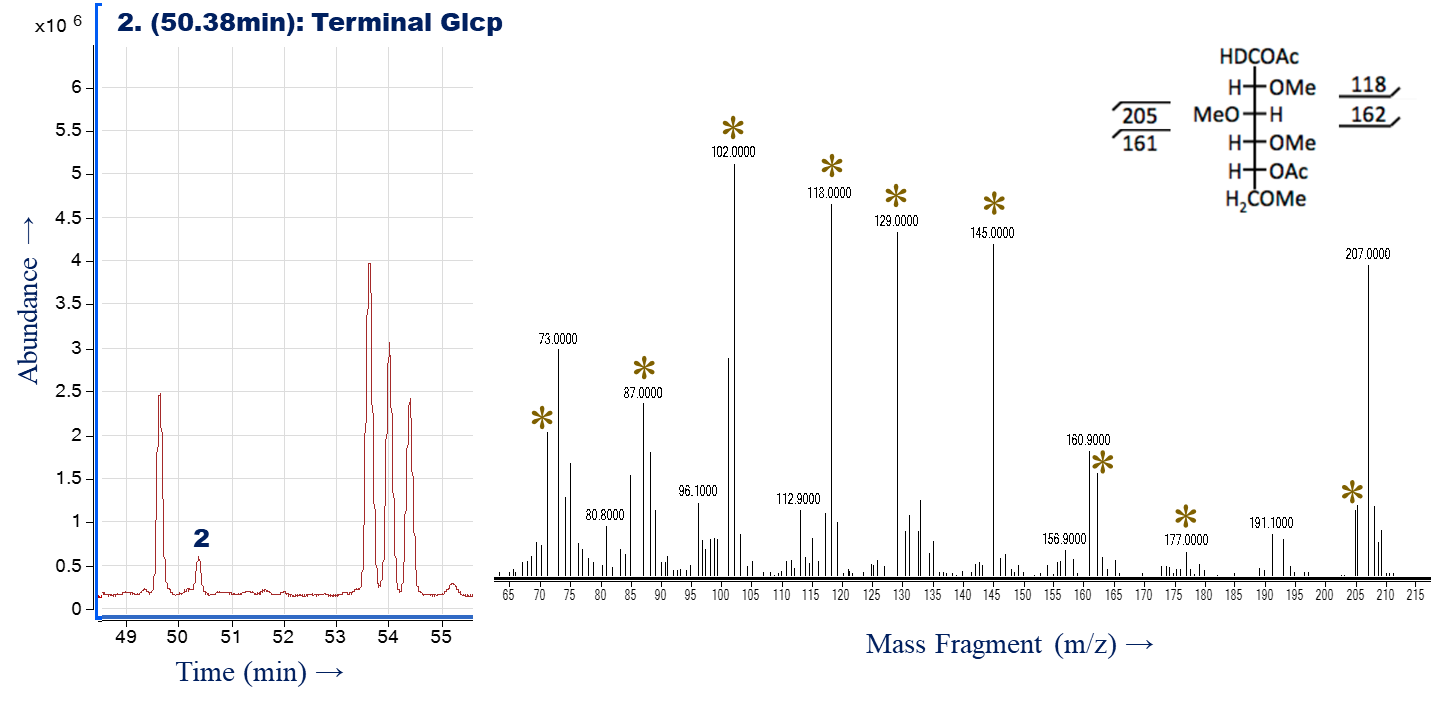


**Sup Fig 3.** The PMAA derivative of terminal-D-glucopyranosyl residue [1,5-Di-O-acetyl-1-deuterio-2,3,4,6-tetra-O-methyl-D-glucitol]


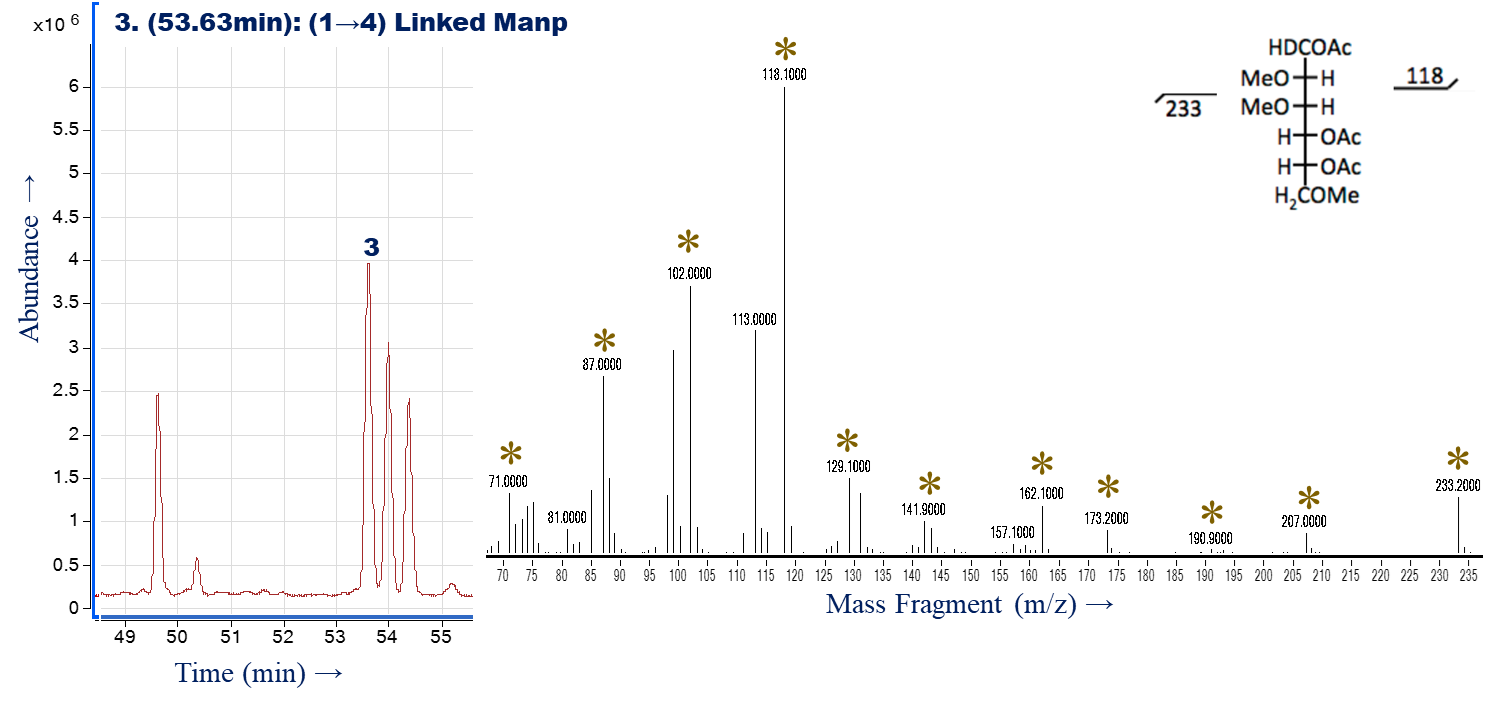


**Sup Fig 4.** The PMAA derivative of a 4-linked-D-mannopyranosyl residue [1,4,5-Tri-O-acetyl-1-deuterio-2,3,6-tri-O-methyl-D-mannitol]


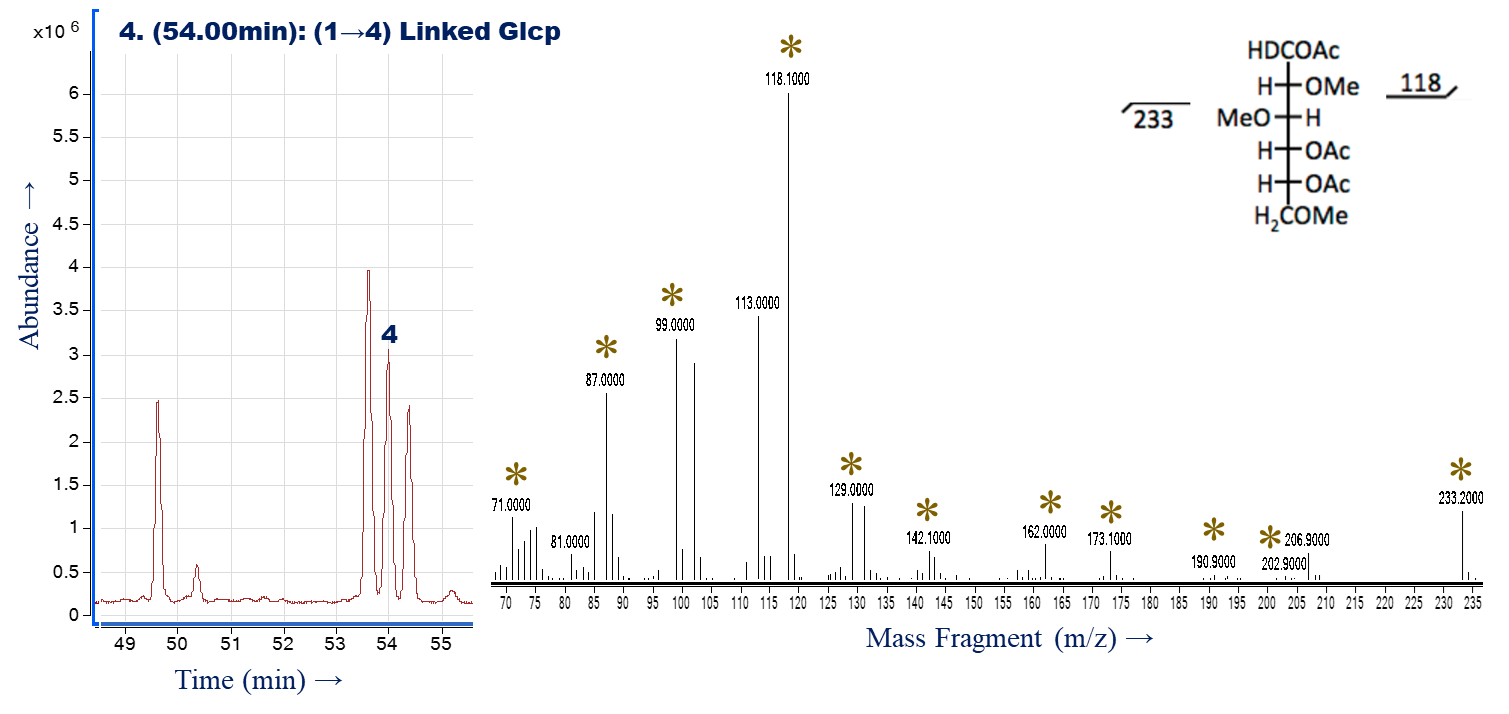


**Sup Fig 5.** The PMAA derivative of 4-linked-D-glucopyranosyl residue [1,4,5-Tri-O-acetyl-1-deuterio-2,3,6-tri-O-methyl-D-glucitol]


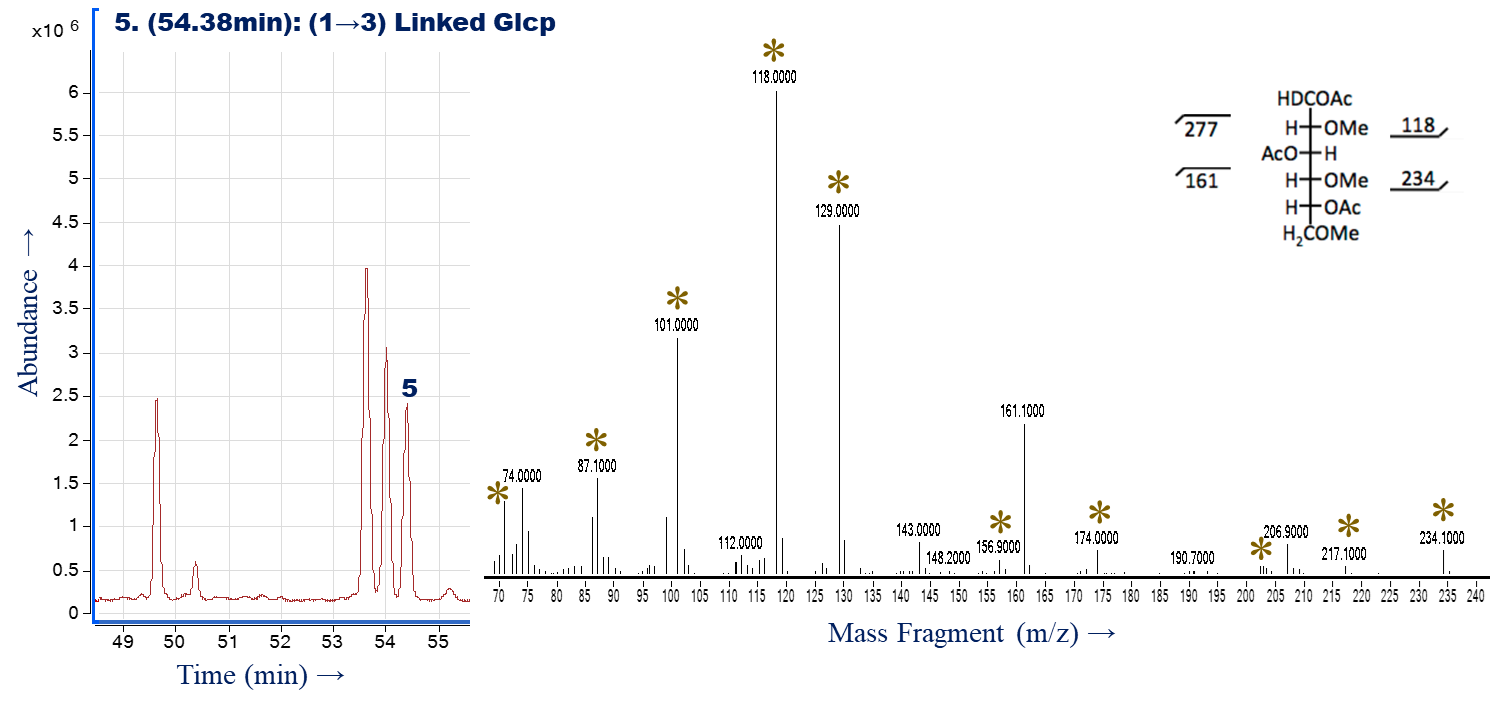


**Sup Fig 6.** The PMAA derivative of 3-linked-D-glucopyranosyl residue [1,3,5-Tri-O-acetyl-1-deuterio-2,4,6-tri-O-methyl-D-glucitol]

**
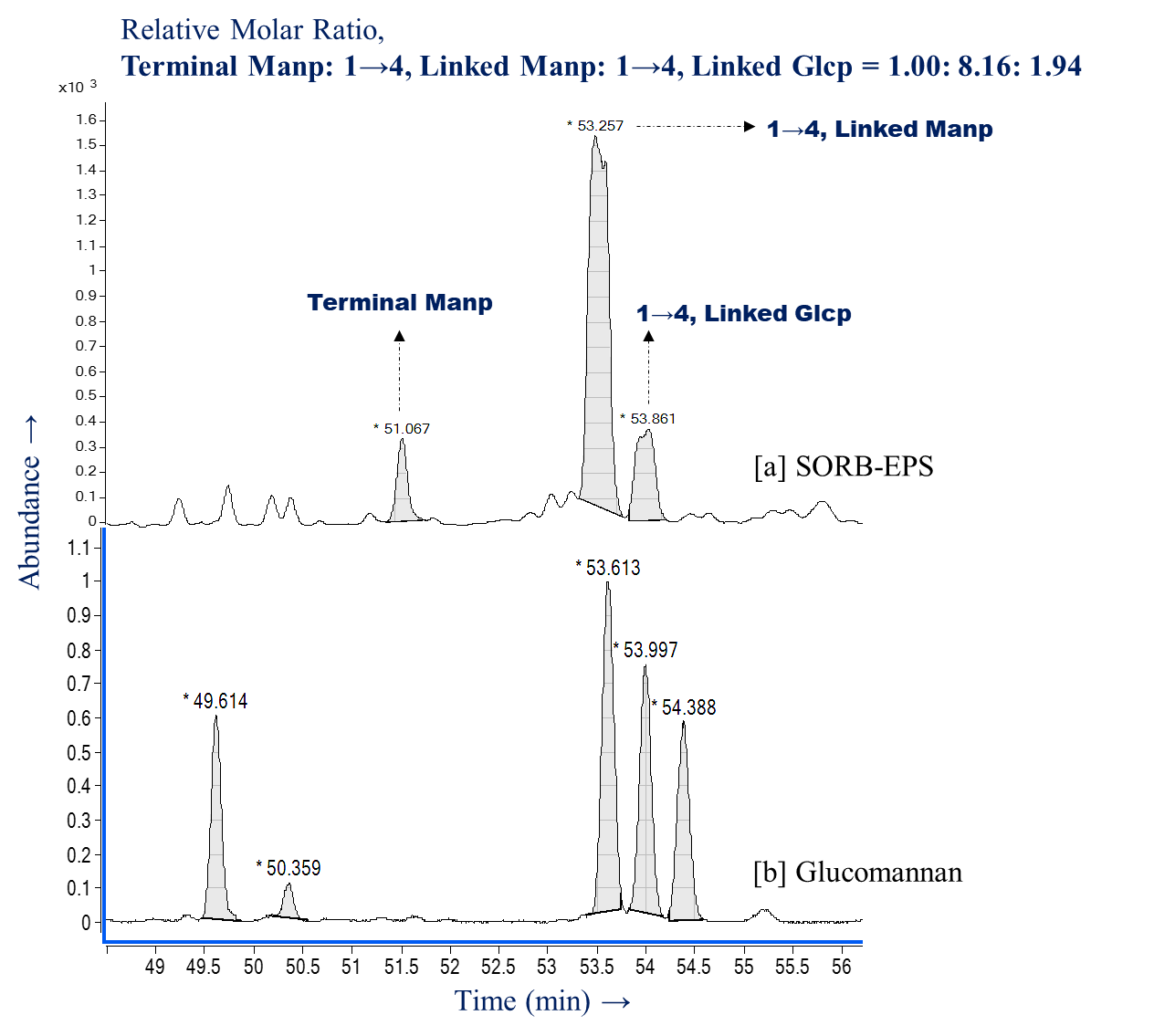
**

**Sup Fig 7.** Gas chromatography-mass spectrometry chromatograms of methylated SORB-EPS [a] and Glucomannan [b].
