# Supplementary material for: Structural insight into a glucomannan-type extracellular polysaccharide produced by a marine Bacillus altitudinis SORB11 from Southern Ocean
Source: Sci Rep. 2022 Sep 29;12:16322. doi: 10.1038/s41598-022-20822-3 (PMC9523031; doi:10.1038/s41598-022-20822-3)
Supplement: Supplementary file 2 — Supplementary Information 2. [file 41598_2022_20822_MOESM2_ESM.docx]

**Supplementary File 2.** Glycosyltransferase family proteins that present in the genome of *B. altitudinis* SORB11.

| **Protein product** | **Start** | **Stop** | **Strand** | **Length** | **Protein Name** | **Template** | **GMQE** | **Seq Identity** | **Description** |
| --- | --- | --- | --- | --- | --- | --- | --- | --- | --- |
| WP_079919164.1  [i] | 6246 | 7514 | - | 422 | glycosyltransferase family 2 protein | 4hg6.1.A | 0.33 | 26.14% | Cellulose Synthase Subunit A [Structure of a cellulose synthase - cellulose translocation intermediate] |
| WP_079919138.1  [ii] | 13962 | 16088 | - | 708 | glycosyltransferase family 39 protein | 5ezm.1.A | 0.32 | 20.42% | 4-amino-4-deoxy-L-arabinose transferase or related glycosyltransferases of PMT family |
| WP_079919300.1  [iii] | 99417 | 101693 | - | 758 | glycosyltransferase | 4p02.1.A | 0.53 | 31.39% | Cellulose Synthase subunit A |
| WP_079920555.1  [iv] | 1567 | 2751 | - | 394 | glycosyltransferase | 4nc9.2.A | 0.48 | 18.94% | GDP-mannose-dependent alpha-(1-2)-phosphatidylinositol mannosyltransferase |
| WP_025208004.1  [v] | 14191 | 15219 | - | 342 | MULTISPECIES: glycosyltransferase | 5hea.1.A | 0.45 | 31.34% | Putative glycosyltransferase (GalT1) |
| WP_017360119.1  [vi] | 131640 | 132410 | - | 256 | MULTISPECIES: glycosyltransferase | 1qgs.1.A | 0.84 | 64.80% | Protein (spore coat polysaccharide biosynthesis protein spsa) UDP-magnesium complex of SPSA |
| WP_025208082.1  [vii] | 30420 | 33119 | + | 899 | MULTISPECIES: glycosyltransferase | 4nc9.2.A | 0.1 | 8.94% | GDP-mannose-dependent alpha-(1-2)-phosphatidylinositol mannosyltransferase |
| WP_017360421.1  [viii] | 41716 | 42906 | + | 396 | MULTISPECIES: glycosyltransferase family 2 protein | 6pxu.1.A | 0.34 | 20.63% | Polypeptide N-acetylgalactosaminyltransferase 12 |
| WP_025208067.1  [ix] | 3113 | 3868 | + | 251 | MULTISPECIES: glycosyltransferase | 5hea.1.A | 0.57 | 29.38% | Putative glycosyltransferase (GalT1) |
| WP_017368088.1  [x] | 17488 | 18327 | - | 279 | MULTISPECIES: glycosyltransferase | 6yv9.2.A | 0.49 | 18.78% | Glycosyl transferase, family 2; Mannosyltransferase in complex with GDP-Man and Mn^2+^ |
| WP_079920540.1  [xi] | 16365 | 17495 | - | 376 | MULTISPECIES: glycosyltransferase family 1 protein | 4nc9.2.A | 0.49 | 20.00% | GDP-mannose-dependent alpha-(1-2)-phosphatidylinositol mannosyltransferase |
| WP_079919219.1  [xii] | 25827 | 27041 | + | 404 | glycosyltransferase family 4 protein | 4nc9.2.A | 0.46 | 14.09% | GDP-mannose-dependent alpha-(1-2)-phosphatidylinositol mannosyltransferase |
| WP_079919634.1  [xiii] | 52243 | 53316 | - | 357 | glycosyltransferase family 2 protein | 7msn.1.A | 0.55 | 22.46% | SPbeta prophage-derived glycosyltransferase SunS |
| WP_035701555.1  [xiv] | 12938 | 13930 | - | 330 | MULTISPECIES: glycosyltransferase family 2 protein | 5ekp.1.A | 0.72 | 43.27% | Uncharacterized glycosyltransferase sll0501 |
| sp\|Q7PC76\|CSLA1_ORYSJ  [xv] | - | - | - | 521 | Glucomannan 4-beta-mannosyltransferase 1 | 4p02.1.A | 0.48 | 22.25% | Cellulose Synthase subunit A |
| WP_079920541.1  [xvi] | 18330 | 19469 | - | 379 | glycosyltransferase family 4 protein | 4nc9.2.A | 0.5 | 18.70% | GDP-mannose-dependent alpha-(1-2)-phosphatidylinositol mannosyltransferase |
| WP_079919220.1  [xvii] | 27038 | 28327 | + | 429 | glycosyltransferase | 1f0k.1.A | 0.41 | 12.46% | UDP-n-acetylglucosamine-n-acetylmuramyl-(pentapeptide) pyrophosphoryl-undecaprenol n-acetylglucosamine transferase |
| WP_035389886.1  [xviii] | 172501 | 173169 | + | 222 | MULTISPECIES: glycosyltransferase family protein | 2qgi.1.A | 0.5 | 15.58% | ATP synthase subunits region ORF 6 [The UDP complex structure of the sixth gene product of the F1-ATPase operon] |
| WP_079920362.1  [xix] | 70471 | 72465 | - | 664 | glycosyltransferase family 39 protein | 5ezm.1.A | 0.33 | 15.92% | 4-amino-4-deoxy-L-arabinose transferase or related glycosyltransferases of PMT family |
| WP_079919082.1  [xx] | 2826 | 4223 | + | 465 | bifunctional glycosyltransferase family 2 protein | 5ndf.5.B | 0.29 | 22.18% | Polypeptide N-acetylgalactosaminyltransferase 2 |
| WP_017360114.1  [xxi] | 126293 | 127033 | - | 246 | MULTISPECIES: glycosyltransferase family protein | 6oew.2.A | 0.71 | 30.47% | Cytidylyltransferase |
| WP_079920543.1  [xxii] | 3837 | 5084 | + | 415 | glycosyltransferase | 3c48.1.A | 0.34 | 14.83% | Predicted glycosyltransferases |
| WP_052320822.1  [xxiii] | 69101 | 70228 | - | 375 | MULTISPECIES: glycosyltransferase | 6h0b.2.A | 0.34 | 18.06% | Polypeptide N-acetylgalactosaminyltransferase 4 [GalNAc-T4 in complex with UDP, manganese and the diglycopeptide 6] |
| WP_045033965.1  [xxiv] | 21221 | 21991 | - | 256 | MULTISPECIES: WecB/TagA/CpsF family glycosyltransferase | 7mpk.1.A | 0.63 | 33.47% | N-acetylglucosaminyldiphosphoundecaprenol N-acetyl-beta-D-mannosaminyltransferase |
| WP_008346430.1  [xxv] | 37900 | 39189 | - | 429 | MULTISPECIES: glycosyltransferase family 2 protein | 1qgs.1.A | 0.42 | 41.15% | Protein (spore coat polysaccharide biosynthesis protein SPSA) |
| WP_007497795.1  [xxvi] | 139173 | 140000 | - | 275 | MULTISPECIES: glycosyltransferase | 5tze.1.A | 0.44 | 20.10% | Glycosyl transferase [in complex with UDP-GlcNAc] |
| WP_008341983.1  [xxvii] | 26592 | 27575 | + | 327 | MULTISPECIES: glycosyltransferase family 2 protein | 5ekp.1.A | 0.7 | 42.30% | Uncharacterized glycosyltransferase sll0501; Structure of the polyisoprenyl-phosphate glycosyltransferase GtrB (WT) |
